# Increased effectiveness of carbon ions in the production of reactive oxygen species in normal human fibroblasts

**DOI:** 10.1093/jrr/rru083

**Published:** 2014-10-10

**Authors:** Till Dettmering, Sebastian Zahnreich, Miriam Colindres-Rojas, Marco Durante, Gisela Taucher-Scholz, Claudia Fournier

**Affiliations:** 1 GSI Helmholtz Centre for Heavy Ion Research, Biophysics, Planckstraße 1, 64291 Darmstadt, Germany; 2 TU Darmstadt, Institute for Condensed Matter Physics, Hochschulstraße 6–8, 64289 Darmstadt, Germany

**Keywords:** oxidative stress, high-LET, fibroblasts, reactive oxygen species, growth arrest, genomic instability

## Abstract

The production of reactive oxygen species (ROS), especially superoxide anions (O_2_^·–^), is enhanced in many normal and tumor cell types in response to ionizing radiation. The influence of ionizing radiation on the regulation of ROS production is considered as an important factor in the long-term effects of irradiation (such as genomic instability) that might contribute to the development of secondary cancers. In view of the increasing application of carbon ions in radiation therapy, we aimed to study the potential impact of ionizing density on the intracellular production of ROS, comparing photons (X-rays) with carbon ions. For this purpose, we used normal human cells as a model for irradiated tissue surrounding a tumor. By quantifying the oxidization of Dihydroethidium (DHE), a fluorescent probe sensitive to superoxide anions, we assessed the intracellular ROS status after radiation exposure in normal human fibroblasts, which do not show radiation-induced chromosomal instability. After 3–5 days post exposure to X-rays and carbon ions, the level of ROS increased to a maximum that was dose dependent. The maximum ROS level reached after irradiation was specific for the fibroblast type. However, carbon ions induced this maximum level at a lower dose compared with X-rays. Within ∼1 week, ROS decreased to control levels. The time-course of decreasing ROS coincides with an increase in cell number and decreasing p21 protein levels, indicating a release from radiation-induced growth arrest. Interestingly, radiation did not act as a trigger for chronically enhanced levels of ROS months after radiation exposure.

## INTRODUCTION

Carbon ions are successfully being used in radiotherapy to inactivate tumors that are untreatable with classical methods using photons [1, 2]. Heavy ions are, in general, more effective than photons in inactivating tumors as a result of higher ionization density, and the surrounding normal tissue is better spared than when using photon irradiation as a result of their inverted depth–dose profile [2]. However, an improved understanding of the physiological consequences of exposure to carbon ions is required, in particular with respect to effects evoked in normal tissue located within the irradiation field.

While ionizing radiation directly damages the DNA in the short term, radiation-induced oxidative stress can damage the DNA chronically and contribute to genomic instability (discussed as a promoter of secondary cancers [3]). Oxidative stress is manifested as an increase in the production of reactive oxygen species (ROS), which arise from a range of cellular sources [4]: a large fraction come from mitochondria in the form of superoxide anions (O_2_^·–^) as a normal physiological process [5]. In addition, upregulation of NADPH oxidases can be the cause of an increase in the production of ROS [6]. The net level of intracellular ROS at a given time is determined by the balance of ROS production and ROS scavenging (through antioxidative systems, e.g. superoxide dismutase, catalase and the glutathione system) [7]. A perturbation of this balance in normal human tissue can result in increased ROS levels and oxidative stress.

For X-rays it has been shown that radiation exposure alters intracellular levels of ROS in human tumor and immortalized cells [8–10]. The reason for this increase is likely mitochondrial damage associated with a change in the membrane potential (reviewed in [11]). Radiation-induced mitochondrial damage causing oxidative stress has moreover been proposed as a trigger for genomic instability [12]. The involvement of ROS in genomic instability has been shown in rodent models after X-ray exposure *in vitro* [13–15] and *in vivo* [16, 17]. In addition, a connection between elevated levels of ROS, an impeded oxidative defense and the accumulation of chromosomal damage has been reported in X-irradiated human tumor cells [10]. In foreskin fibroblasts (AG1522), however, an increased long-term chromosomal instability was not observed in our previous studies [18, 19]. Therefore, we addressed the question of whether increased ROS levels occur in AG1522 fibroblasts that do not show chromosomal instability.

Furthermore, we sought to assess the impact of carbon ions on ROS production. In this context, we used normal human skin fibroblasts as a cell type always present in the radiation field during radiotherapy. We compared carbon ions and X-rays for their ability to increase ROS levels acutely within days after irradiation, as well as chronically months post exposure. For these investigations, we used doses that are in the range of doses absorbed by the normal tissue for one fraction during radiotherapy. In addition, we tested whether a radiation-induced proliferation block is observed concomitantly to acutely enhanced ROS levels, as suggested by other studies [10]. We found that, in AG1522 cells, intracellular levels of ROS were increased, then decreased again to control levels within one week. The time-course of decreasing ROS levels was accompanied by a release from growth arrest. Although this was similar for both radiation types, the dose–response curves revealed that carbon ions were significantly more efficient compared with X-rays. However, neither X-ray nor carbon ion irradiation was able to trigger chronically enhanced levels of ROS months after radiation exposure.

## MATERIALS AND METHODS

### Cell culture

Normal human neonatal foreskin fibroblasts AG1522 and VH7 were obtained from the Coriell Institute (Camden, NJ, USA) and DKFZ (Heidelberg, Germany), respectively, and normal human fetal lung fibroblasts (IMR-90) were obtained from Cell Systems (St Katherinen, Germany). The cumulative numbers of population doublings were 21–26 (AG1522), 5 (VH7) and 30–33 (IMR-90) at the beginning of the experiments. Cells were grown in Eagle's Modified Essential Medium (Lonza, Cologne, Germany) supplemented with 10% fetal bovine serum (Biochrom, Berlin, Germany) and 1% penicillin/streptomycin (Biochrom) in a 37°C humidified incubator with 95% air/5% CO_2_. For long-term experiments, cells were passaged every 14 days. Medium was replaced every 3 days. All cells were tested negative for mycoplasma contamination by PCR.

### Exposure to X-rays and carbon ions

Cells were exposed to X-rays (250 kV, 16 mA, 1.5 Gy min^−1^). Carbon ions (9.8 MeV u^−1^, 170 keV μm^−1^) were obtained at the universal linear accelerator (UNILAC) facility (GSI, Darmstadt, Germany). To compare the same physical doses, 0.5 Gy was selected for X-rays and carbon ions. To compare the doses at iso-survival levels, we selected the doses of 6 Gy for X-rays and 2 Gy for carbon ions, which we (and others) had determined previously [20, 21]. The corresponding dataset for clonogenic survival of AG1522 cells measured are included in Suppl. Fig. 2. The fluences of carbon ions were 1.8 and 7.35 × 10^6^ particles cm^−2^ for 0.5 and 2 Gy, respectively.

Confluent cells were exposed either to X-rays or carbon ions as described elsewhere [18]. Briefly, prior to irradiation, the culture medium was exchanged with serum-free medium in order to avoid cell cycle stimulation during irradiation. Immediately after irradiation, the medium was replaced with the medium that had been on the cells beforehand. The whole irradiation procedure took ∼30 min, during which the cells were kept at room temperature. After 24 h, the cells were reseeded at a density of 4000 cells cm^–2^ in 25 cm^2^ flasks. The cell numbers were determined using a Coulter Z3 cell counter (Coulter, Sinsheim, Germany).

### Flow cytometric measurement of intracellular ROS levels and p21

**Fig. 1.**
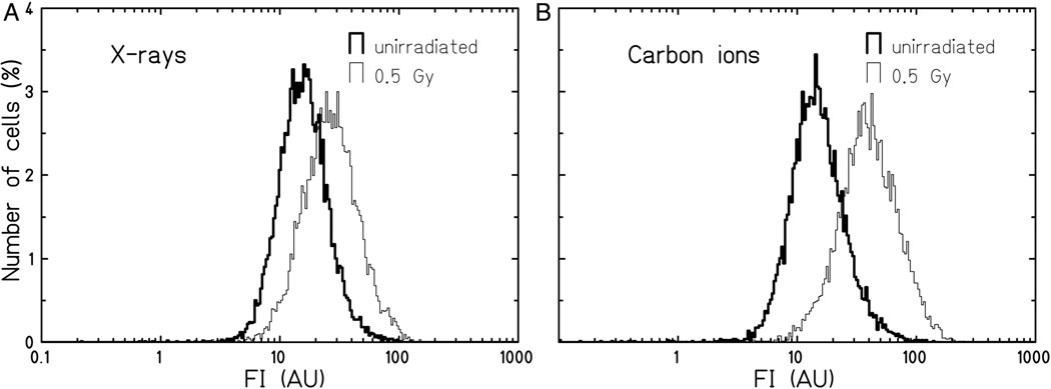
Distributions of DHE fluorescence intensities (FI; arbitrary units, AU) in AG1522 cells 3 days after X-ray (**A**) or carbon ion (**B**) irradiation with 0.5 Gy. The number of cells as percentage of all cells is shown as a function of the fluorescence intensity.

For measurements of ROS, cells were trypsinized, washed and resuspended at a concentration of ∼10^5^ cells ml^–1^ in 4 μM Dihydroethidium (DHE; AnaSpec, Fremont, CA, USA) dissolved in PBS. The measurements of long-term cultures were performed after cells were passaged for maintenance. DHE is reactive mainly, but not exclusively, with superoxide anions, which are mostly generated as a result of electron leakage in the respiratory chain [22]. According to the reported range of between 15 and 30 min [10, 23, 24], an incubation time of 15 min was chosen (longer incubation times did not change staining intensities). After incubation, cells were analyzed in a Partec PAS III flow cytometer (Partec, Münster, Germany). A total of 30 000 cells were analyzed per sample. The mean fluorescence intensity (MFI) was calculated for each sample from the fluorescence intensity histograms (an example is shown in Fig. 1), using the FloMax software (Partec). The relative fluorescence intensity value (RFI) was calculated by dividing the MFI of the irradiated samples by the MFI of the control samples.

For the measurement of p21 protein expression, cells were fixed on each measurement day with 4% paraformaldehyde and stored at 4°C until the end of the experiment. For immunostaining, cells were permeabilized with Triton-X100 (Roth, Karlsruhe, Germany; 0.5% in PBS) and incubated with mouse-anti-p21 antibody (BD Biosciences, Heidelberg, Germany; Cat. No. 610234; 1:100 dilution) for 1 h at room temperature (RT). A goat-anti-mouse antibody conjugated to Alexa Fluor 488 (Life Technologies, Darmstadt, Germany; Cat. No. A-11001; 1:400 dilution) was then added and incubated 45 min at RT. Cells were analyzed in a BD FACSCanto II flow cytometer (BD Biosciences). The fluorescence intensity histograms were established, and the MFI was calculated from the whole histograms, thus including any subpopulations. The flow cytometry-based p21 quantification was validated by a parallel Western Blot detection at the timepoint of 1 day post ionizing irradiation, using the same antibody, and confirming a comparable range of p21 induction of 2.9 ± 0.9-fold and 4.0 ± 1.3-fold for immunofluorescence and Western blot analysis, respectively (triplicate measurements).

### Data analysis

All experiments were performed in triplicates and repeated at least three times, unless stated otherwise. Mean and SEM of all available experiments are given per datapoint, unless stated otherwise. Student's *t*-test with unequal variances was performed to prove significance; two-tailed *P*-values are given. For dose–response curves, a hyperbolic function was fitted against the data:

$$f\;(D)=\frac{L_{\max}\cdot D}{D_{50}+D}+1,$$

D represents the dose, L_max_ represents the maximum ROS level, and D_50_ represents the dose at half maximum ROS level. The equation is a simple hyperbolic model that includes the L_max_ and D_50_. This model is here applied to relative fluorescence intensities (RFIs), normalized to 0 Gy. As a consequence, the RFI for 0 Gy is equal to 1; this value has to be added to the equation.

**Fig. 2.**
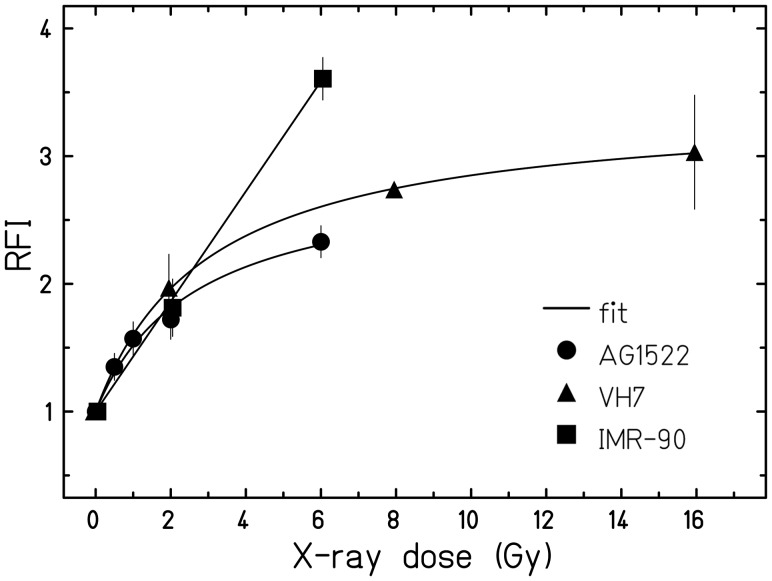
Dose-dependence of ROS levels comparing different fibroblasts (measured by staining with DHE). The fluorescence intensities relative to unirradiated cells (RFI) are shown as a function of the X-ray dose for AG1522 (*n* = 6), VH7 and IMR-90 (both *n* = 1). Plotted are the maximum fluorescence intensities for each dose, typically observed between 3 and 5 days after exposure (see ‘Data analysis’ for details; the corresponding maximum levels can be inferred from Fig. 4).

**Fig. 3.**
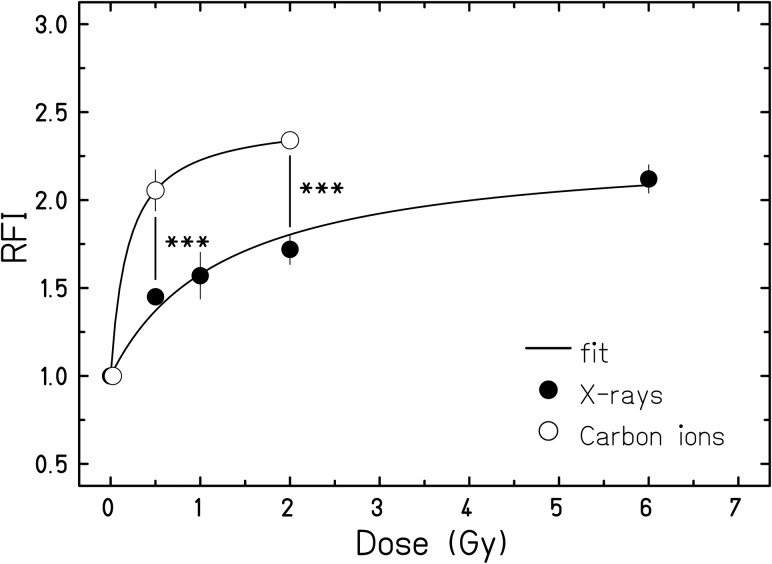
Effect of radiation quality on the dose-dependence of ROS levels in AG1522 cells. RFI (DHE fluorescence intensities relative to unirradiated cells) are shown as a function of dose for carbon ion irradiation (*n* = 3) or X-rays (*n* = 6, data from Fig. 2). The maximum fluorescence intensities for each dose, typically obtained between 3 and 5 days after exposure, were used in the dose–response plot (see ‘Data analysis’ for details). Significant differences between the same physical doses of X-rays and carbon ions; three asterisks indicate *P* < 0.001.

**Fig. 4.**
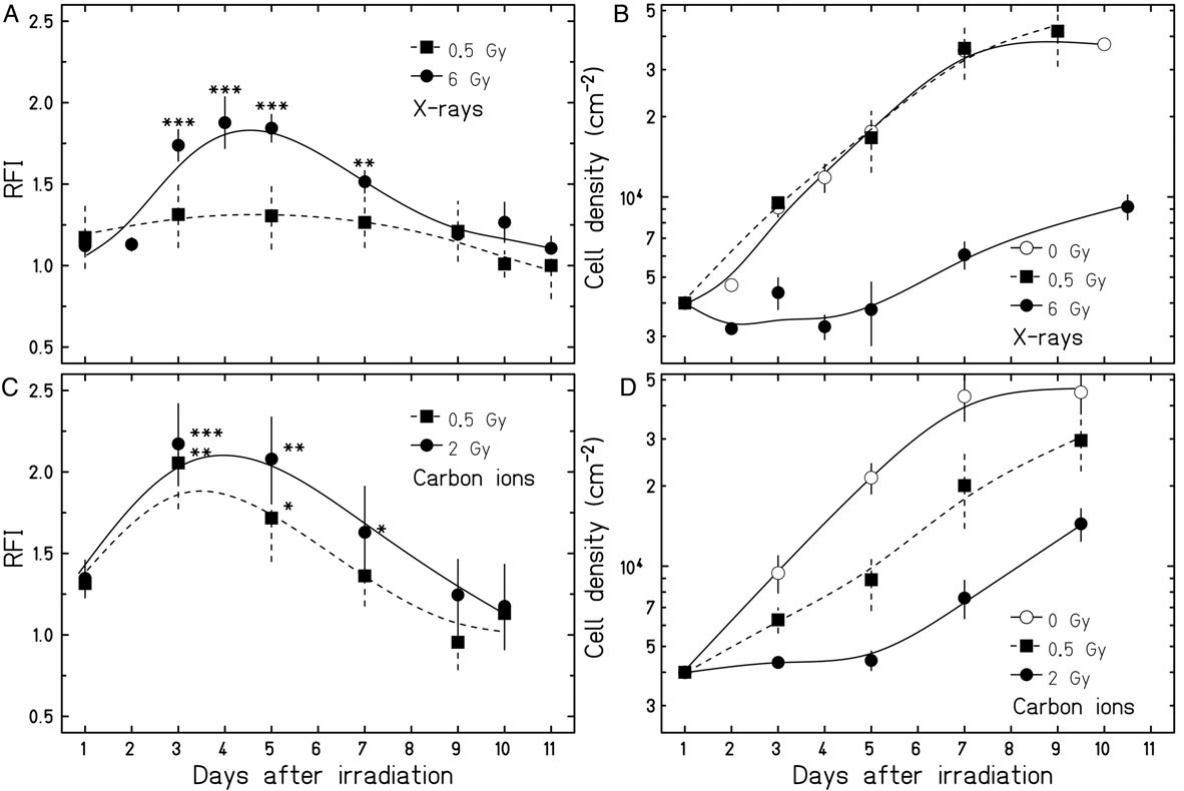
ROS levels and corresponding cell densities of AG1522 cells after X-ray (**A**, **B**; *n* = 3–8) or carbon ion irradiation (**C**, **D**; *n* = 3) as a function of the time after irradiation. Relative fluorescence intensities (RFIs) of DHE normalized to the mean of unirradiated cells (A, C) are shown. In (B) and (D) the corresponding cell densities are depicted. Significant difference of RFI from 0 Gy is indicated by a single asterisk (*P* < 0.05), two asterisks (*P* < 0.01) or three asterisks (*P* < 0.001). Note that the single experiments are averaged over the timepoints, resulting in error bars and significances that are different from Fig. 3 (see ‘Data analysis’ for details).

We followed two separate approaches regarding the data analysis: To compare the dose responses (Figs 2 and 3), we averaged the maximum RFIs reached in the individual experiments over the dose. This step was necessary because the maximum RFIs were occurring at different times after irradiation in each experiment (between 3 and 5 days). To compare the time-course of the ROS levels (Fig. 4), we averaged the RFIs over each timepoint.

## RESULTS

### Intracellular levels of ROS increased with dose within days after X-ray and carbon ion irradiation in a range of fibroblasts

We first examined whether an increase in the DHE fluorescence intensity indicating the presence of ROS is detectable in AG1522 cells within days after exposure to X-rays or carbon ions. Figure 1 shows a representative ﬂow cytometric measurement of the distributions of DHE ﬂuorescence intensities detected in AG1522 ﬁbroblasts 3 days after irradiation with 0.5 Gy X-rays (A) or carbon ions (B). For both radiation qualities, all irradiated cells showed a shift towards higher ﬂuorescence intensities compared with the unirradiated cells.

Aiming to compare the radiation-induced ROS levels in AG1522 cells with other fibroblasts of different origin (VH7 and IMR-90), we next exposed all fibroblast cell lines to X-ray doses of up to 16 Gy (Fig. 2). Note that for each dose the ROS levels plotted in the response curve correspond to the maximum fluorescence intensity values, which were obtained between days 3 and 5 after exposure (see ‘Data analysis’ for details). A comparable increase in ROS levels with dose was observed in all cells up to 2 Gy, showing at this dose an ∼2-fold higher level compared with unirradiated cells. In AG1522 and VH7 cells at 6 or 8 Gy, respectively, ROS were increased ∼2.5-fold compared with control cells, and for VH7 cells a similar level of ROS was also found when higher doses were measured, revealing a saturation of the response. However, a steeper increase in ROS levels was observed in irradiated IMR-90 cells (nearly 4-fold at 6 Gy compared with control cells) suggesting cell-specific effects.

### Carbon ions induce ROS with a similar time-course but more efficiently compared with X-ray irradiation

To assess the influence of radiation quality we additionally examined the ROS levels induced by carbon ions in AG1522 cells (Fig. 3). As in Fig. 2, the plotted maximum fluorescence intensities for each dose were obtained between 3 and 5 days post exposure (see ‘Data analysis’ for details). We observed that carbon ions induced significantly higher levels of ROS than X-rays, comparing the same physical doses of 0.5 and 2 Gy (three asterisks, indicating *P* = 5.9 × 10^–4^ and *P* = 1.5 × 10^−6^, respectively). In addition, a more than 2-fold increase in ROS levels compared with unirradiated cells was observed for the isosurvival doses of 2 Gy carbon ions and 6 Gy X-rays that result in a similar fraction of surviving cells. Interestingly, this shows that the maximum ROS levels reached in AG1522 cells are in the same range for both radiation qualities, in spite of different physical doses.

Next we investigated the time-course of the ROS production after X-ray and carbon ion irradiation. In Fig. 4A and C, the intracellular levels of ROS are plotted over the course of 11 days or 10 days after exposure to the same physical doses (0.5 Gy) of X-rays and carbon ions and isosurvival doses (6 Gy and 2 Gy, respectively). The maximum increase in the ROS level was reached 3–5 days after irradiation for both radiation qualities. In cells irradiated with the high isosurvival dose (6 Gy X-rays, 2 Gy carbon ions), the ROS levels increased up to 2-fold compared with the unirradiated cells at the respective timepoints (X-rays: *P* = 0.0005; carbon ions: *P* = 0.0011). At a later time, the ROS levels returned to the level of the controls and were not significantly increased 1 week post irradiation. It should be noticed that the time-course of ROS production was similar for both radiation types, despite the different dose response.

### The decrease in ROS levels coincides with a release from growth arrest and a decline in p21 protein levels

A relation between ROS level and release from growth arrest after radiation exposure has been reported for tumor cells [10]. Previous studies in human fibroblasts [25], and also in AG1522 cells [18], have shown a rapid cell cycle inhibition after irradiation. According to these results, a large part of the AG1522 cells are irreversibly arrested after exposure to very high doses ( >8 Gy of X-rays), but for lower doses a majority of the cells are released and reenter the cell cycle during the first week after irradiation. In order to see whether increased levels of ROS coincide with growth inhibition and whether the subsequent decrease in the ROS levels overlaps with the release from the radiation-induced growth delay, we determined the development of cell densities over time in parallel with the ROS measurements. Figure 4B and D show the corresponding cell densities for the same samples analyzed in Fig. 4A and C. While irradiation with 0.5-Gy X-rays did not result in a growth arrest, exposure to 0.5-Gy carbon ions clearly induced a growth inhibition. Exposure to higher doses (6-Gy X-rays or 2-Gy carbon ions) led to a growth arrest in the majority of the cells lasting at least over 5 days. After this period of time the growth arrest was released, and ROS declined to the level of control cells.

As a next step, we investigated in parallel the temporal pattern of ROS levels and the protein p21 (CDKN1A), known as a radiation-induced inhibitor of the cell cycle [26]. The signal intensities in X-irradiated cells relative to the controls, shown in Table 1, reveal that on day 1, p21 protein was induced, whereas the corresponding ROS levels remained at control level. However, on day 3 and during the following days, both p21 and ROS levels were elevated in the irradiated cells before declining to control levels afterwards. Note that on day 11 (determined only for X-rays), the relative p21 amount in the irradiated cells was even below 1.0, most likely because the p21 amount was higher in the control cells compared with the previous timepoints. This effect has been reported previously and is most likely related to a quiescent state due to contact inhibition in the unirradiated cell culture [27, 28].

**Table 1.** Relative ROS and p21 fluorescence intensities compared with controls for different timepoints after irradiation in AG1522 cells

Irradiation	*n*	Days after irradiation	ROS	SEM	*n*	p21	SEM	*n*
X-rays (6 Gy)	2	1	1.0		1	2.1		1
		3	1.6	0.2	3	1.7	0.1	3
		4	2.1	0.2	3	1.5	0.3	3
		5	1.7	0.2	6	1.6	0.2	6
		7	1.5	0.1	6	2.0	0.2	6
		10	1.5	0.1	2	1.7	0.1	3
		11	1.1	0.0	3	^a^0.5	0.1	3
Carbon ions (2 Gy)	1	3	2.4	0.3	3	4.0		2
		5	1.6	0.1	3	1.9	0.2	2
		7	1.2	0.2	2	1.5	0.2	2
		9	1.0	0.4	2	1.0		1

^a^Controls were confluent.

**Fig. 5.**
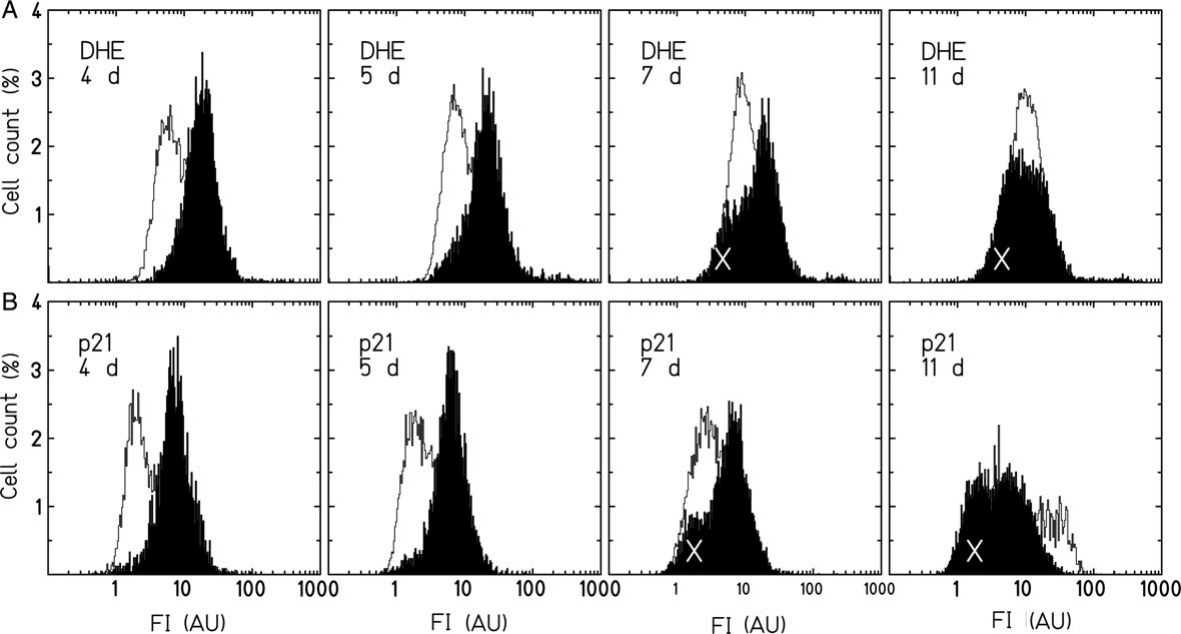
Representative distributions of DHE (**A**) or p21 (**B**) fluorescence intensities in AG1522 cells 4, 5, 7 and 11 days after irradiation with X-rays (6 Gy, filled histograms; 0 Gy, blank histograms). The number of cells as a percentage of all cells is shown as a function of the fluorescence intensity. ‘X’ indicates an emerging subpopulation that demonstrated reduced p21 or DHE fluorescence intensities.

In Fig. 5A and B, the histograms of the respective fluorescence intensities for ROS and p21 demonstrate that with the decline of ROS between days 5 and 7 after exposure, subpopulations with a lower amount of ROS and p21 emerge concomitantly (indicated by ‘X’). Note that in the same time-frame, the first cells start to proliferate (Fig. 4B and D).

### ROS levels are not enhanced many generations after radiation exposure

**Fig. 6.**
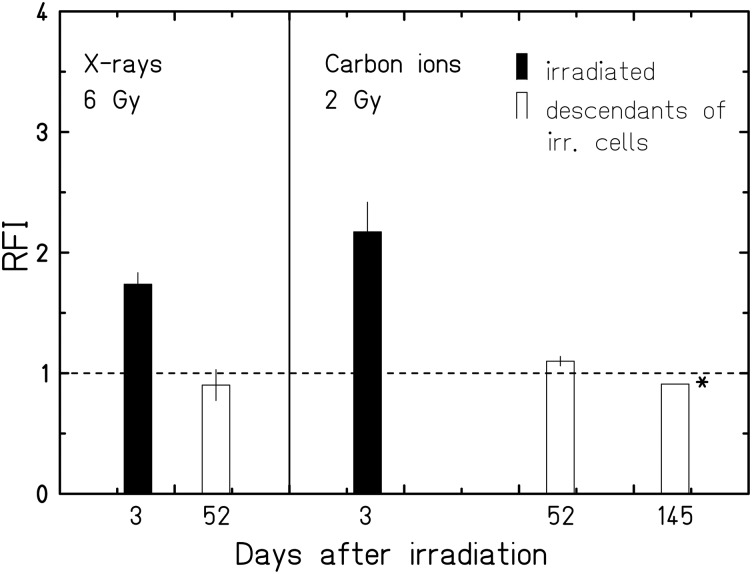
ROS levels in the descendant cells of irradiated AG1522 cells. Shown is the relative fluorescence intensity (RFI) of DHE at an early and a late timepoint after exposure. Filled bars indicate irradiated cells (*n* = 7 for 6 Gy X-rays; *n* = 3 for 2 Gy carbon ions); open bars indicate the descendants (*n* = 2 (duplicates), * single measurement) of cells irradiated with 6 Gy X-rays or 2 Gy carbon ions. The dashed horizontal line indicates the level of unirradiated cells at the respective timepoint.

To investigate whether ROS levels are only elevated during the first week or if they are elevated across many generations after exposure, we cultured irradiated AG1522 fibroblasts for 52 and 145 days and measured the intracellular levels of ROS. Based on earlier results [20], only 2–3% of the irradiated AG1522 cells are still able to form colonies after exposure to the indicated doses, hence the fraction of irradiated cells after 52 or 145 days is negligible. As demonstrated in Fig. 6, the relative ROS levels were not increased compared with unirradiated cells at these late timepoints after irradiation, regardless of the radiation quality.

Taken together, the results presented here show differences between fibroblasts of different origin in the extent of radiation-induced accumulation of ROS. Furthermore, higher radiation doses induce higher levels of ROS, but the dose response of the VH7 and AG1522 cells indicate a saturation of intracellular ROS levels after irradiation. Carbon ion irradiation turned out to be more effective at inducing ROS than X-rays when comparing the same physical doses in AG1522 fibroblasts.

## DISCUSSION

In the present work we used normal human fibroblasts (AG1522) to assess the oxidative response during the first days after exposure to different types of ionizing radiation. After photon irradiation, the fluorescence intensity distributions for the entire population were shifted to higher values and the mean ROS levels increased with dose, reaching a maximum between 3 and 5 days and declining to control level within 7 days (Fig. 1A, 4A). The oxidization of DHE was used as an indicator of intracellular ROS, including the measurement of superoxide anions, which likely originate from mitochondria [12]. We also found an increase in intracellular ROS levels by measuring the Dichlorofluorescein probe after X-irradiation (DCF; Suppl. Fig. 1), which supports the results found with DHE. This is in good agreement with reported data from earlier studies using normal and immortalized human fibroblasts [8, 24].

We also compared AG1522 cells to other normal fibroblasts and observed increasing ROS levels in all cells several days post irradiation (Fig. 2). For each of the different fibroblasts, the ROS increment did not exceed a certain maximum, suggesting that it could be specific to different donors or to the tissue of origin of the fibroblasts. This supports the notion that not only the primary radiation event, but also cell-specific factors may participate in the modulation of the increment in ROS production several days post irradiation. To consider a possible correlation between ROS production and cell killing of the different fibroblast strains, we have compiled published and added new data on clonogenic survival [20, 29] (Suppl. Fig. 2). The levels of radiosensitivities are similar for IMR-90 and VH7 cells and moderately different for AG1522 cells. Although the clear dose and LET dependence of ROS production is suggestive of a connection to clonogenic survival, the different behavior of the ROS dose responses observed in the studied cells does not reflect a simple relation to cell killing.

The particular focus in the present study was to investigate a potential difference between the effects of photons and carbon ions on the intracellular level of ROS. The normal human fibroblasts chosen for this work (AG1522 cells) have been widely used to assess the radiation response for various endpoints after carbon ion exposure in comparison with photon exposure [19, 30–32]. The increase in ROS levels in AG1522 cells was also detectable for carbon irradiation with a similar time-course (Fig. 4C), but already at a lower dose compared with X-rays (Fig. 3). A more than 2-fold increase in ROS production was observed following 2 Gy of carbon ions, whereas 6 Gy of photons were needed to obtain the same effect. At this level of effect, the relative biological effectiveness (RBE) of carbon ions is ∼3, and is in the same range as the RBE values reported for DNA damage-related effects, i.e. chromosomal damage, survival, accelerated differentiation, and senescence [33]. A possible explanation for the higher efficiency of carbon ions in increasing cellular ROS levels could be a stronger impairment of the anti-oxidative capacities of the exposed cells, as has been reported recently for normal human fibroblasts exposed to isosurvival doses of photons and carbon ions [21]. Interestingly, protons were found to be equally effective, but high-energy iron ions with similar ionizing density to carbon ions were found to be less effective in ROS production compared with X-rays [34]. This study was conducted in epithelial cells, and it cannot be decided whether the physical difference in the track structure of iron versus carbon ions are the basis for the discrepancy, or the different response of epithelial cells versus fibroblasts.

It should be noticed that, in AG1522 cells, a clearly lower dose of carbon ions (0.5 Gy) leads to a ROS induction only slightly below the maximum (Fig. 3), indicating an even higher RBE at lower doses of carbon ions. Thus, the higher LET of carbon ions compared with photons seems to modify the dose response, but not the maximum ROS levels, probably due to the aforementioned cell-specific maximum for the response observed for various fibroblasts.

It has been previously reported that ROS play an important role in apoptosis induction [35], in particular in precancerous cells [36], or at least coincide with the induction of apoptosis in IMR-90 fibroblasts [24]. However, we did not observe an increased frequency of apoptosis in AG1522 cells within 8 days after exposure (Suppl. Table 1), indicating that the increased ROS levels in these cells are not related to apoptosis.

The decline in intracellular levels of ROS occurred concomitantly with a release from the radiation-induced growth arrest, regardless of the radiation quality (Fig. 4B and D), suggesting that intracellular ROS levels and the release from arrest are interrelated. In an earlier study, we observed a rapid accumulation and induction of the cell cycle-regulating proteins p53 and p21 (more pronounced) within a few hours after irradiation [18, 37], thus preceding the increment of ROS that was measurable in the present study. This argues for a p53/p21-mediated growth arrest after irradiation without a direct influence of elevated ROS on the *onset* of the proliferation block. Nonetheless, a transition to growth arrest in response to chemically induced oxidative stress is well known in normal cells [38–40], and in X-irradiated tumor cells. In tumor cells, a *release* from growth arrest was observed to coincide with decreasing ROS production [10]. Moreover, a causal relationship between ROS and p21 was suggested by overexpression of p21; after scavenging ROS, the cells were released from cell cycle arrest and proliferation was resumed [41]. In our study, the gradual release from growth arrest shown was reflected by an emerging subpopulation of cells, which demonstrated a simultaneous decline in ROS levels and p21 protein amounts (Fig. 5). Based on the assumption that the proliferation rate of this subpopulation is similar to the controls, a rough estimate of the fraction of cells reentering the cell cycle after day 4 reveals that 30% of the cells irradiated with 6-Gy X-rays were released from cell cycle arrest and continued to proliferate. This percentage approximately corresponds to the subpopulation of cells with low ROS and p21 protein level at day 7. However, based on our data we cannot determine if there is a causal relationship between ROS and p21 levels, or if other factors determine the release from growth arrest and the decrease in intracellular ROS levels occurs coincidentally to the onset of proliferation.

For the long-term fate of irradiated cells, it is relevant whether ROS are enhanced in the cell generations descending from the irradiated cells. Chronically increased ROS levels have mostly been reported for various cell types or rodent tissues that demonstrate an intrinsic genomic instability [14, 42], or become genomically unstable after X-ray [17, 43] or high-LET exposure [6]. This indicates both the occurrence and persistence of chromosomal instability, and elevated ROS levels strongly depend on the model system used, for cells i.e. on the cell type. Susceptibility for manifesting chromosomal instability may be different in normal human fibroblasts of different origin. In lung fibroblasts (IMR-90), we have indication of a high spontaneous occurrence of DNA and chromosomal damage (our own unpublished results) and an accumulation of cytogenetic damage at the end of their life span [44]. In a previously published study, we used AG1522 cells to show that no radiation-induced genomic instability was observed in addition to the increasing instability of ageing fibroblasts [19]. In contrast to a more pronounced chromosomal instability reported for human lymphocytes after α-particle compared with X-ray exposure [45], we found that exposure to carbon ions compared with X-rays did not have a major impact on cytogenetic aberrations occurring in the descendants of irradiated fibroblasts [18]. Consistently, we demonstrated in the present study that ROS levels were not chronically enhanced in the descendants of normal human fibroblasts, representing a genomically *stable* cell system, irrespective of the radiation quality (Fig. 6).

## SUPPLEMENTARY DATA

Supplementary data are available at the *Journal of Radiation Research* online.

## FUNDING

This work was supported by the Bundesministerium für Bildung und Forschung (02S8497 and 02NUK017A), Forschungsinstitut Bad Gastein, and the Helmholtz Graduate School for Heavy Ion Research (HGS-HIRe). Funding to pay the Open Access publication charges for this article was provided by GSI.

## Supplementary Material

Supplementary Data

## References

[1] Durante M, Loeffler JS (2010). Charged particles in radiation oncology. Nat Rev Clin Oncol.

[2] Schardt D, Elsässer T, Schulz-Ertner D (2010). Heavy-ion tumor therapy: physical and radiobiological benefits. Rev Mod Phys.

[3] Hanahan D, Weinberg RA (2011). Hallmarks of cancer: the next generation. Cell.

[4] Wen X, Wu J, Wang F (2013). Deconvoluting the role of reactive oxygen species and autophagy in human diseases. Free Radic Biol Med.

[5] Liu Y, Fiskum G, Schubert D (2002). Generation of reactive oxygen species by the mitochondrial electron transport chain. J Neurochem.

[6] Datta K, Suman S, Kallakury BV (2012). Exposure to heavy ion radiation induces persistent oxidative stress in mouse intestine. PLoS ONE.

[7] Dröge W (2002). Free radicals in the physiological control of cell function. Physiol Rev.

[8] Kobashigawa S, Suzuki K, Yamashita S (2011). Ionizing radiation accelerates Drp1-dependent mitochon- drial fission, which involves delayed mitochondrial reactive oxygen species production in normal human fibroblast-like cells. Biochem Biophys Res Comm.

[9] Saenko Y, Cieslar-Pobuda A, Skonieczna M (2013). Changes of reactive oxygen and nitrogen species and mitochondrial functioning in human K562 and HL60 cells exposed to ionizing radiation. Radiat Res.

[10] Tulard A, Hoffschir F, de Boisferon FH (2003). Persistent oxidative stress after ionizing radiation is involved in inherited radiosensitivity. Free Radic Biol Med.

[11] Kam WWY, Banati RB (2013). Effects of ionizing radiation on mitochondria. Free Radic Biol Med.

[12] Kim GJ, Chandrasekaran K, Morgan WF (2006). Mitochondrial dysfunction, persistently elevated levels of reactive oxygen species and radiation-induced genomic instability: a review. Mutagenesis.

[13] Limoli CL, Giedzinski E, Morgan WF (2003). Persistent oxidative stress in chromosomally unstable cells. Cancer Res.

[14] Samper E, Nicholls DG, Melov S (2003). Mitochondrial oxidative stress causes chromosomal instability of mouse embryonic fibroblasts. Aging Cell.

[15] Tominaga H, Kodama S, Matsuda N (2004). Involvement of reactive oxygen species (ROS) in the induction of genetic instability by radiation. J Radiat Res.

[16] Clutton SM, Townsend KM, Walker C (1996). Radiation-induced genomic instability and persisting oxidative stress in primary bone marrow cultures. Carcinogenesis.

[17] Wang Y, Liu L, Pazhanisamy SK (2010). Total body irradiation causes residual bone marrow injury by induction of persistent oxidative stress in murine hematopoietic stem cells. Free Radic Biol Med.

[18] Fournier C, Winter M, Zahnreich S (2007). Interrelation amongst differentiation, senescence and genetic instability in long-term cultures of fibroblasts exposed to different radiation qualities. Radiother Oncol.

[19] Fournier C, Zahnreich S, Kraft D (2012). The fate of a normal human cell traversed by a single charged particle. Sci Rep.

[20] Fournier C, Scholz M, Weyrather WK (2001). Changes of fibrosis-related parameters after high- and low-LET irradiation of fibroblasts. Int J Radiat Biol.

[21] Laurent C, Leduc A, Pottier I (2013). Dramatic increase in oxidative stress in carbon-irradiated normal human skin fibroblasts. PLoS ONE.

[22] Wardman P (2007). Fluorescent and luminescent probes for measurement of oxidative and nitrosative species in cells and tissues: progress, pitfalls, and prospects. Free Radic Biol Med.

[23] Narayanan P, Goodwin E, Lehnert B (1997). Alpha particles initiate biological production of superoxide anions and hydrogen peroxide in human cells. Cancer Res.

[24] Rugo RE, Secretan MB, Schiestl RH (2002). X radiation causes a persistent induction of reactive oxygen species and a delayed reinduction of TP53 in normal human diploid fibroblasts. Radiat Res.

[25] Di Leonardo A, Linke SP, Clarkin K (1994). DNA damage triggers a prolonged p53-dependent G1 arrest and long-term induction of Cip1 in normal human fibroblasts. Genes Dev.

[26] Bartek J, Lukas J (2001). Mammalian G1- and S-phase checkpoints in response to DNA damage. Curr Opin Cell Biol.

[27] Perucca P, Cazzalini O, Madine M (2009). Loss of p21 CDKN1A impairs entry to quiescence and activates a DNA damage response in normal fibroblasts induced to quiescence. Cell Cycle.

[28] Takeuchi S, Takahashi A, Motoi N (2010). Intrinsic cooperation between p16INK4a and p21Waf1/Cip1 in the onset of cellular senescence and tumor suppression *in vivo*. Cancer Res.

[29] Ohno T, Nishimura T, Nakano K (1984). Differential recovery from potentially lethal damage in normal human lung fibroblasts after irradiation with ^60^Co gamma-rays and accelerated N-ion beam. Int J Radiat Biol Relat Stud Phys Chem Med.

[30] Esposito G, Belli M, Campa A (2006). DNA fragments induction in human fibroblasts by radiations of different qualities. Radiat Prot Dosimetry.

[31] Kawata T, Gotoh E, Durante M (2000). High-LET radiation-induced aberrations in prematurely condensed G2 chromosomes of human fibroblasts. Int J Radiat Biol.

[32] Wu H, Furusawa Y, George K (2002). Analysis of unrejoined chromosomal breakage in human fibroblast cells exposed to low- and high-LET radiation. J Radiat Res.

[33] Tenhumberg S, Gudowska-Nowak E, Nasonova E (2007). Cell cycle arrest and aberration yield in normal human fibroblasts. II: Effects of 11 MeV u-1 C ions and 9.9 MeV u-1 Ni ions. . *Int J Radiat Biol*.

[34] Wan XS, Bloch P, Ware JH (2005). Detection of oxidative stress induced by low- and high-linear energy transfer radiation in cultured human epithelial cells. Radiat Res.

[35] Simon HU, Haj-Yehia A, Levi-Schaffer F (2000). Role of reactive oxygen species (ROS) in apoptosis induction. Apoptosis.

[36] Portess DI, Bauer G, Hill MA (2007). Low-dose irradiation of nontransformed cells stimulates the selective removal of precancerous cells via intercellular induction of apoptosis. Cancer Res.

[37] Fournier C, Wiese C, Taucher-Scholz G (2004). Accumulation of the cell cycle regulators TP53 and CDKN1A (p21) in human fibroblasts after exposure to low- and high-LET radiation. Radiat Res.

[38] Chen Q, Fischer A, Reagan JD (1995). Oxidative DNA damage and senescence of human diploid fibroblast cells. Proc Natl Acad Sci U S A.

[39] Dumont P, Burton M, Chen QM (2000). Induction of replicative senescence biomarkers by sublethal oxidative stresses in normal human fibroblast. Free Radic Biol Med.

[40] Luo Y, Zou P, Zou J (2011). Autophagy regulates ROS-induced cellular senescence via p21 in a p38 MAPKalpha dependent manner. Exp Gerontol.

[41] Macip S, Macip S, Igarashi M (2002). Inhibition of p21-mediated ROS accumulation can rescue p21- induced senescence. EMBO J.

[42] Limoli CL, Giedzinski E (2003). Induction of chromosomal instability by chronic oxidative stress. Neoplasia.

[43] Dayal D, Martin SM, Owens KM (2009). Mitochondrial complex II dysfunction can contribute significantly to genomic instability after exposure to ionizing radiation. Radiat Res.

[44] Zahnreich S, Krunic D, Melnikova L (2012). Duplicated chromosomal fragments stabilize shortened telomeres in normal human IMR-90 cells before transition to senescence. J Cell Physiol.

[45] Kadhim MA, Marsden SJ, Goodhead DT (2001). Long-term genomic instability in human lymphocytes induced by single-particle irradiation. Radiat Res.

