# Supplementary material for: Increased effectiveness of carbon ions in the production of reactive oxygen species in normal human fibroblasts
Source: J Radiat Res. 2014 Oct 10;56(1):67–76. doi: 10.1093/jrr/rru083 (PMC4572590; doi:10.1093/jrr/rru083)
Supplement: Supplementary Data [file supp_56_1_67__index.html]

Increased effectiveness of carbon ions in the production of reactive oxygen species in normal human fibroblasts — Supplementary Data 

# Increased effectiveness of carbon ions in the production of reactive oxygen species in normal human fibroblasts

## Supplementary Data

Supplementary Data

**Files in this Data Supplement:**

- Supplementary Data - Docx file
