# Supplementary material for: Increased effectiveness of carbon ions in the production of reactive oxygen species in normal human fibroblasts
Source: J Radiat Res. 2014 Oct 10;56(1):67–76. doi: 10.1093/jrr/rru083 (PMC4572590; doi:10.1093/jrr/rru083)

**Supplementary Data**

Table S1: Apoptosis rate in AG1522 cells determined using the Annexin-V assay

| Days after irradiation | X-ray dose (Gy) | No. of cells investigated | % Apoptotic |
| --- | --- | --- | --- |
| 4 | 0 | 1400 | 0.4 |
|  | 6 | 1034 | 0.9 |
| 8 | 0 | 1023 | 0.4 |
|  | 6 | 1070 | 0.2 |

Supplementary Figure 1: Distributions of Dichlorofluorescein (DCF) fluorescence intensities in AG1522 fibroblasts 3 days after X-irradiation with 6 Gy. The number of cells in percent of all cells is shown as a function of the fluorescence intensity. The increased fluorescence intensity observed after irradiation using DHE (Figure 1) is also visible with DCF.


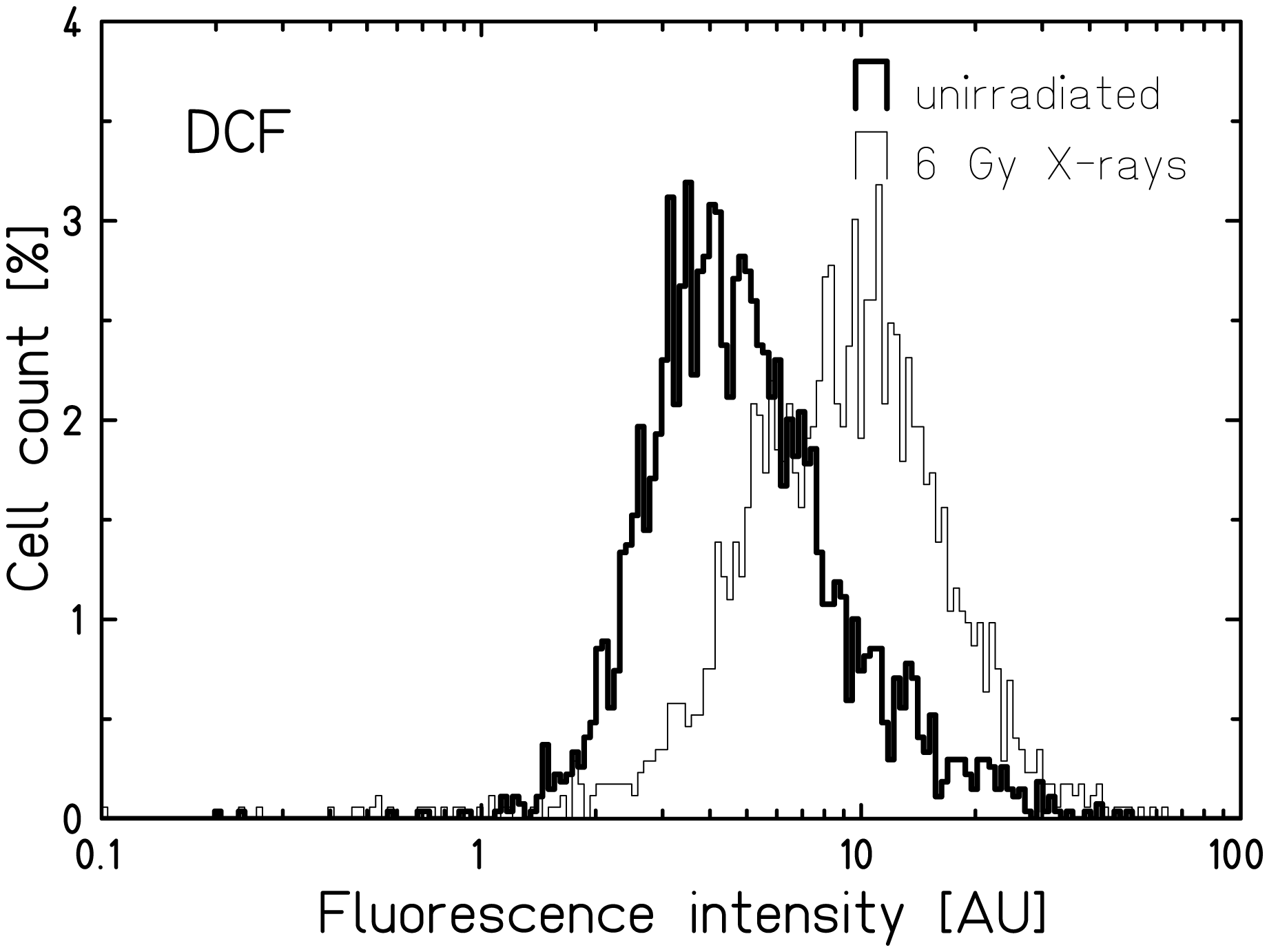


Supplementary Figure 2: Clonogenic survival of the different fibroblasts used in this study. As indicated, carbon ion data for AG1522 (11 MeV/u) and γ-ray data for IMR-90 cells are redrawn from [20] and [29], respectively. Clonogenic survival assay was performed as described in [20], but cells were replated 24h after X-ray exposure according to the ROS measurement. All data points have been measured at least in triplicates.


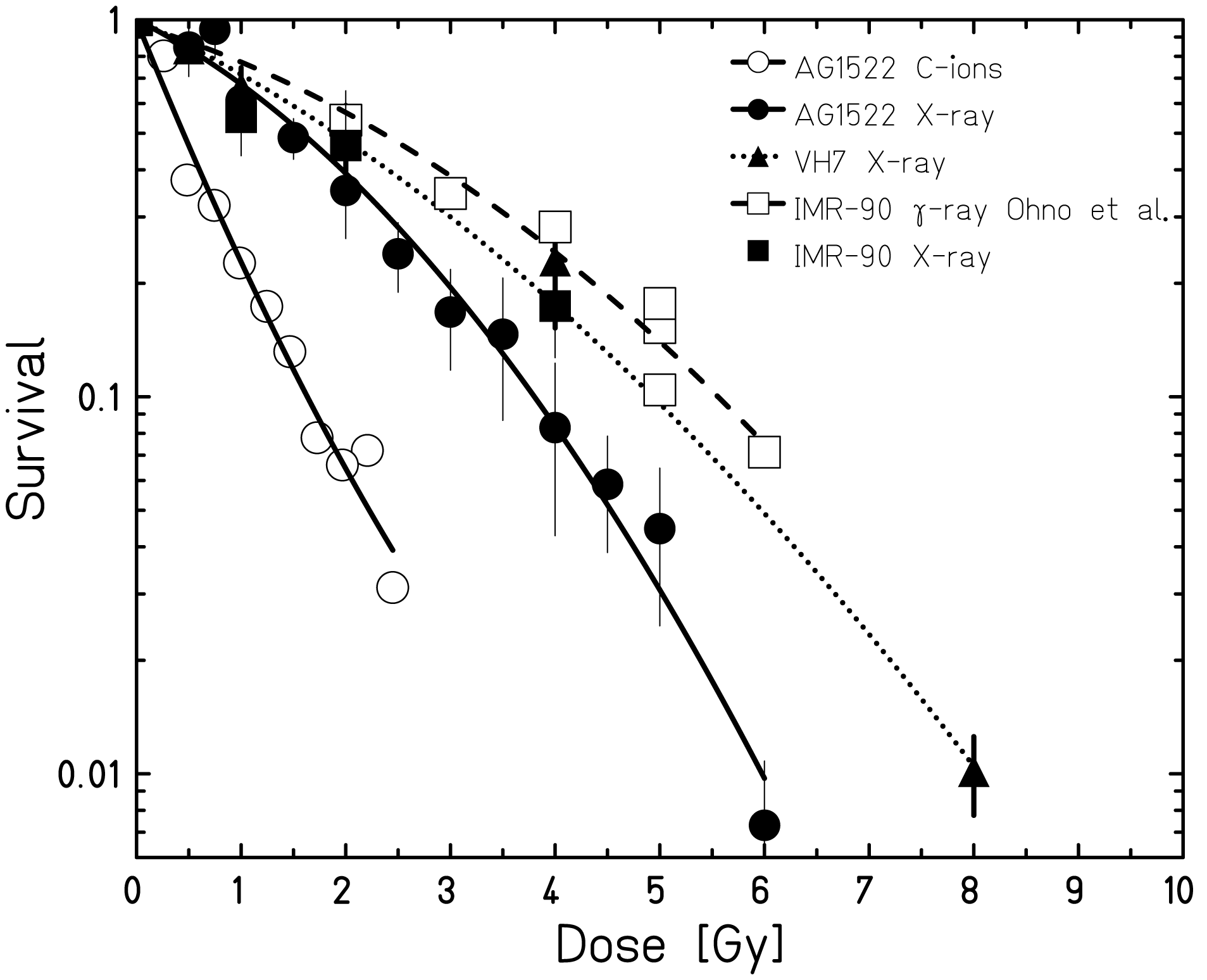

Supplement: Supplementary Data [file supp_rru083_rru083supp.docx]
